# Evaluation of Virulence Factors and Antibiotic Sensitivity Pattern of Escherichia Coli Isolated from Extraintestinal Infections

**DOI:** 10.7759/cureus.604

**Published:** 2016-05-09

**Authors:** Ritu Vaish, MSS Pradeep, CR Setty, Venkataramana Kandi

**Affiliations:** 1 Department of Microbiology, Prathima Institute of Medical Sciences; 2 Department of Microbiology, Dr. Pinnamaneni Siddhartha Institute of Medical Sciences and Research Foundation

**Keywords:** escherichia coli, virulence determinants, antimicrobial susceptibility patterns, hemolysin production, cell surface hydrophobicity, serum resistance, gelatinase production, extraintestinal infections

## Abstract

**Introduction:**

Identification of virulence determinants among the clinically isolated microorganisms assumes greater significance in the patient management perspective. Among the hospitalized patients, extremes of age groups (neonatal and geriatric age patients), patients who are debilitated due to other associated medical conditions, patients taking immunosuppressive therapy, and patients undergoing major surgeries are prone to infections with previously nonpathogenic or opportunistic pathogens. Screening of the pathogenic potential of such bacteria and identifying their virulence factors and antimicrobial susceptibility patterns could be instrumental in better patient care and management.

**Materials & methods:**

In this study, we evaluated the virulence determinants and antimicrobial susceptibility patterns of 100 clinical isolates of *E. coli *collected from extraintestinal infections and 50 control strains of *E. coli*. Hemolysin production, serum resistance, cell surface hydrophobicity, and gelatinase production were tested using standard laboratory procedures.

**Results:**

Results showed that *E. coli*strains have a variable pattern of virulence markers that included hemolysin production (9%), cell surface hydrophobicity (9%), serum resistance (93%), and gelatinase production (2%). Antimicrobial susceptibility testing revealed a higher rate of resistance against cephalothin (84%) and ampicillin (98%). Susceptibility to amikacin (80%) and co-trimoxazole (47%) was variable and none of the test strains revealed resistance to imipenem. The control strains in contrast exhibited fewer virulence factors and the least resistance to antibiotics.

**Conclusion:**

In conclusion, the study results revealed that *E. coli* isolated from extraintestinal infections had demonstrated greater virulence and higher resistance to antibiotics as compared to the *E. coli* strains isolated from healthy individuals.

## Introduction

*Escherichia coli *(*E. coli*) is a gram negative and rod-shaped bacteria belonging to the phylum *Proteobacteria* and family *Enterobacteriaceae*. They are present in the intestinal tract of both human and animals as a commensal [1]. Although most *E. coli* species are harmless, few strains have been associated with a variety of human infections that include and are not limited to urinary tract infections, wound infections, bacteremia, meningitis, and other soft tissue infections [2-3]. *E. coli* uses its virulence factors and the debilitated condition of the individuals and could be responsible for mild to invasive infections including hospital-acquired infections.

Among the various infections caused by *E. coli, *urinary tract infections (UTI) assume greater significance as observed by the fact that *E. coli* is responsible for more than 90% of UTI cases. Due to its easy transmissibility and the presence of virulence determinants, the normal human and animal intestinal colonizers invade the urinary tract through the ascending route and cause UTI [4].

As with many other microbial infections, human clinical isolates of *E. coli *have a relatively high potential for developing antibiotic resistance. Occurrence and spread of extended spectrum beta-lactamase (ESBL) and carbapenemase producing *E. coli* strains should be considered as a serious concern. Infections caused by *E. coli* NDM-1 gene (New Delhi metallo beta-lactamase) producers and the multi-drug resistant strains are a threat in the hospital as infections caused by such bacteria are difficult to treat. Their ubiquitous nature and their versatile habitats; their presence in human and animal as commensals, isolated from the environment (water); and their pathogenic potential to cause various infections usually among immunocompromised people makes E. coli a true opportunistic pathogen [5].

*E. coli* is a gram-negative, rod-shaped, motile, non-sporing, lactose fermenting and facultatively anaerobic bacterium belonging to the genus *Escherechia*. *E. coli* is present as a normal colonizer in the lower intestinal tract of humans benefiting the host by producing vitamins that include vitamin K. *E. coli* are usually transmitted to humans through feco-oral route and are responsible for diarrhea owing to the presence of various enterotoxins. The ability of *E. coli* to cause extraintestinal infections depends largely on a combination of several virulence factors, which help the *E. coli* survive under adverse conditions present in those sites [2].

The virulence factors contributing to the colonization and pathogenicity of *E. coli* include adhesins (function like hemagglutinin by helping to adhere uroepithelial cells), serum resistance, hemolysin production, cell surface hydrophobicity, resistance to phagocytosis, production of cytotoxic necrotizing factor, K1 antigen, siderophore, gelatinase production, and others [2]. The present study aims to evaluate the virulence determinants (hemolysin production, serum resistance, cell surface hydrophobicity, and gelatinase production) demonstrated by the clinical isolates of *E. coli* responsible for extraintestinal infections and their antimicrobial susceptibility patterns.

## Materials and methods

This study included 100 isolates of *E. coli* collected from extraintestinal infections from both inpatients and outpatients attending Dr. Pinnamaneni Siddhartha Institute of Medical Sciences and Research Foundation (Dr. PSIMS & RF) and was performed in the department of microbiology, between August 2011 and December 2012. Informed consent was obtained from the patients for this study.

Specimens collected included urine, pus, blood, cerebrospinal fluid, sputum, and synovial fluid. The samples were processed using standard bacteriological procedures and isolates were identified based on gram staining, colony morphology on blood agar, MacConkey agar, and by standard and conventional biochemical tests. Further, all the strains were assessed for antibiotic susceptibility patterns against commonly used antibiotics. Fifty isolates of *E. coli *from stool samples from apparently healthy individuals who had visited the hospital for routine health checkup formed the control group. The isolates were maintained by inoculating onto semi solid nutrient agar butts and stored at 2-4°​C until further detection.

### Detection of virulence factors

Hemolysin production: Hemolysin is a cytolytic toxin, a protein secreted by some *E. coli* isolates, which is also referred to as alpha-hemolysin. Production of alpha-hemolysin was demonstrated by using blood agar plate hemolysis method. Clinical strains of *E. coli* were inoculated onto 5% sheep blood agar and incubated overnight at 35°​C. Hemolysin production was detected by the presence of a zone of complete clearance of erythrocytes around the colony as observed against transmitted light [6-7].

Cell surface hydrophobicity (CSH)/Salt aggregation test (SAT): Bacteria were tested for their hydrophobic property by using different molar concentrations of ammonium sulphate. *E. coli *grown on nutrient agar plates were inoculated into 1 ml of phosphate buffered saline (PBS) at pH 6.8. The turbidity was matched with McFarland standard 6-7 to finally give a colony count of 5x10^9^ colonies/ml. Different molar concentrations of ammonium sulphate (0.625 M, 1.25 M, and 2.5 M) were prepared. On a clean and grease-free glass slide, 10 µl of bacterial suspension prepared in PBS was mixed with equal volumes of ammonium sulphate solution at different molarity and rocked for a minute to observe for clumping. *E. coli* strains that had SAT value ≤ 1.25 M were considered hydrophobic and those which demonstrated aggregates with salt particles by forming clumps were considered as positive for cell surface hydrophobicity. The highest dilution of ammonium sulphate solution producing a visible clumping was treated as a positive titer for salt aggregation test [8-9].

Serum resistance: Overnight growth of *E. coli* on 5% sheep blood agar was suspended in PBS to produce a count of 2.5x10^4 ^cfu/ml. 10 µl of the suspension was then inoculated on 5% sheep blood agar plates. In a test tube 50 µl of bacterial suspension is mixed with 50 µl of human serum and is incubated for a period of 180 minutes. After incubation 10 µl of this suspension was inoculated on 5% blood agar and incubated overnight at 37°​C and the viable count was determined. Susceptibility of bacteria to serum bactericidal activity was expressed as the percentage of bacteria surviving after 180 minutes in relation to the original count of bacteria determined at 0 minutes. Strains were considered as serum sensitive if the viable count dropped by 1% of the initial value and resistant if > 90% of organisms survived after 180 minutes [10-11].

Gelatinase production/protease activity: Gelatinase activity was demonstrated using gelatin agar. The gelatin agar plate was inoculated with *E. coli* and was incubated at 37°​C for 24 hours. After incubation, the plates were flooded with mercuric chloride solution. Development of the zone of opacity surrounding the colonies was considered positive for gelatinase production [2].

Antibiotic susceptibility testing: The antibiotic susceptibility testing was performed using Kirby-Bauer disk diffusion method in accordance with Clinical and Laboratory Standards Institute (CLSI) guidelines. The antibiotic discs (Hi Media, Mumbai, India) tested included ampicillin (10 µg), cephalothin (30 µg), ceftriaxone (30 µg), gentamicin (10 µg), amikacin (30 µg), nitrofurantoin (300 µg), norfloxacin (10 µg), ciprofloxacin (5 µg), cotrimoxazole (1.25/ 23.75 µg) and imipenem (10 µg) [12].

After overnight incubation at 37°​C, the diameter of the zone of inhibition around the antibiotic disk was measured in millimeter scale from the under-surface of the plate. Depending on the standard zone sizes for each antibiotic, the result was interpreted as sensitive, intermediate, or resistant.

## Results

Out of 100 isolates of *E. coli*, 65 strains were isolated from urine, 29 from pus, one from liver abscess, two from endotracheal secretion, and one from gastric lavage as shown in Table 1.

**Table 1:** Specimen-wise Distribution of Isolates

Nature of Specimen	Number
Urine	65
Vaginal swab	2
Liver abscess aspirate	1
Pus swab	29
Endotracheal tube	2
Gastric lavage	1
Total	100

The underlying medical conditions identified among the subjects' group from whom the extraintestinal isolates of *E. coli* were collected included patients suffering from diabetes mellitus, uterine prolapse, liver abscess, hypertension, stress, urinary incontinence, retention of urine, cerebrovascular accident (CVA) with hemiparesis, cancer of cervix, uterine fibroids, catheterization, benign prostatic hypertrophy, hypospadias, and dysplastic kidney.

Among the virulence factors studied, hemolysin production (9%), cell surface hydrophobicity (9%), serum resistance (93%), and gelatinase production (2%) were noted for all the isolates as detailed in Table 2. 

**Table 2:** Virulence Factors Studied in Different Clinical and Control Isolates

Specimen Source	Hemolysin Production	Cell surface Hydrophobicity	Serum Resistance	Gelatinase Production
Pus	1	4	25	1
Urine	7	5	62	1
High vaginal swab	1	0	2	0
Liver abscess aspirate	0	0	1	0
ET tube	0	0	2	0
Gastric lavage	0	0	1	0
Total	9 (9%)	9 (9%)	93 (93%)	2 (2%)

**Figure 1:**
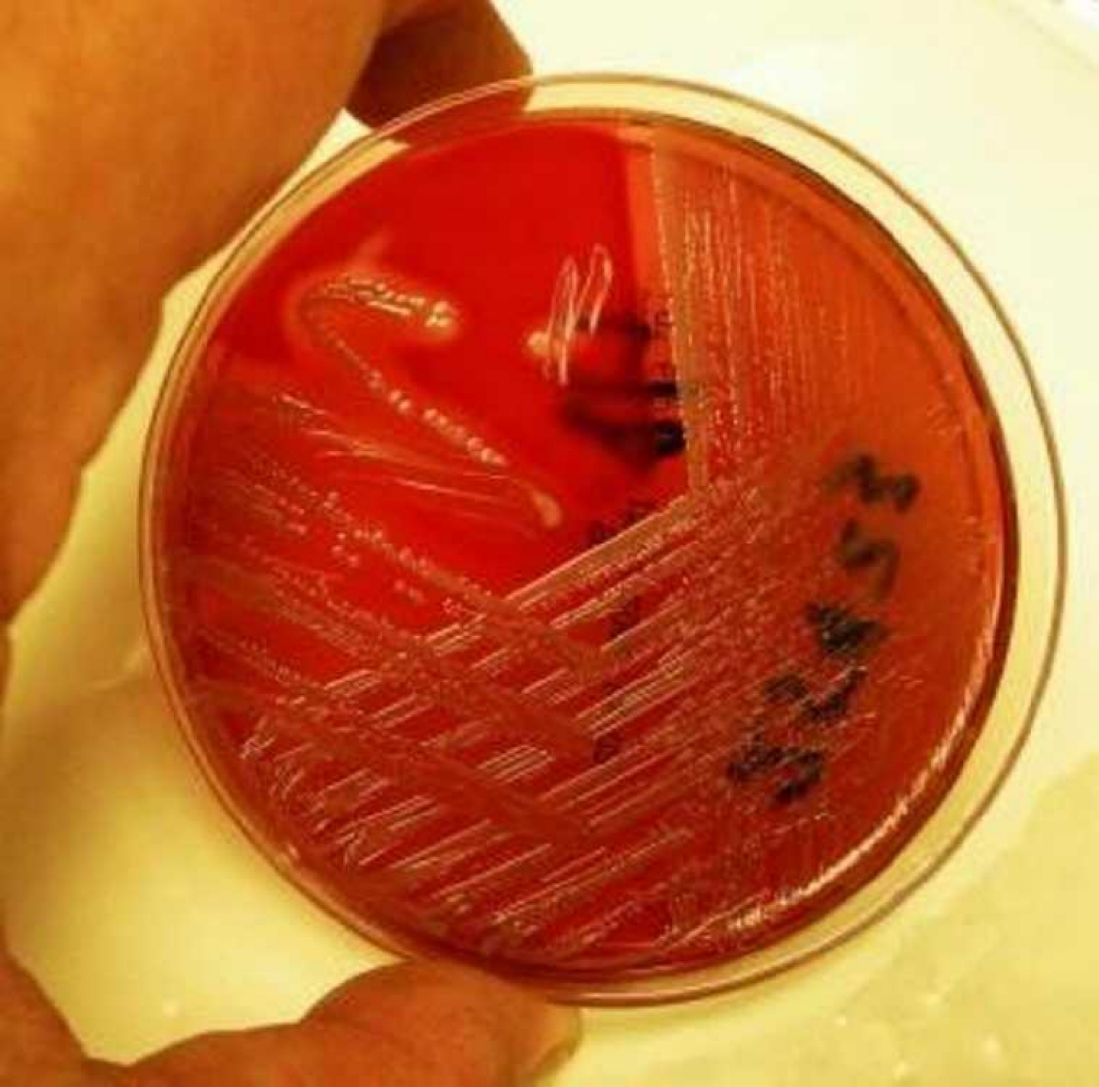
Picture Depicting Hemolytic activity on Blood Agar

The most common virulence factor identified was serum resistance (93%). Cell surface hydrophobicity and gelatinase production were observed in the least number of strains as shown in Table 2. Comparison of the virulence determinants demonstrated among the test groups and the controls revealed that hemolysin production was observed in 47% of *E. coli *isolates in the study group, whereas all control groups isolates were negative for hemolysin production as shown in Figure 1. 

**Figure 2:**
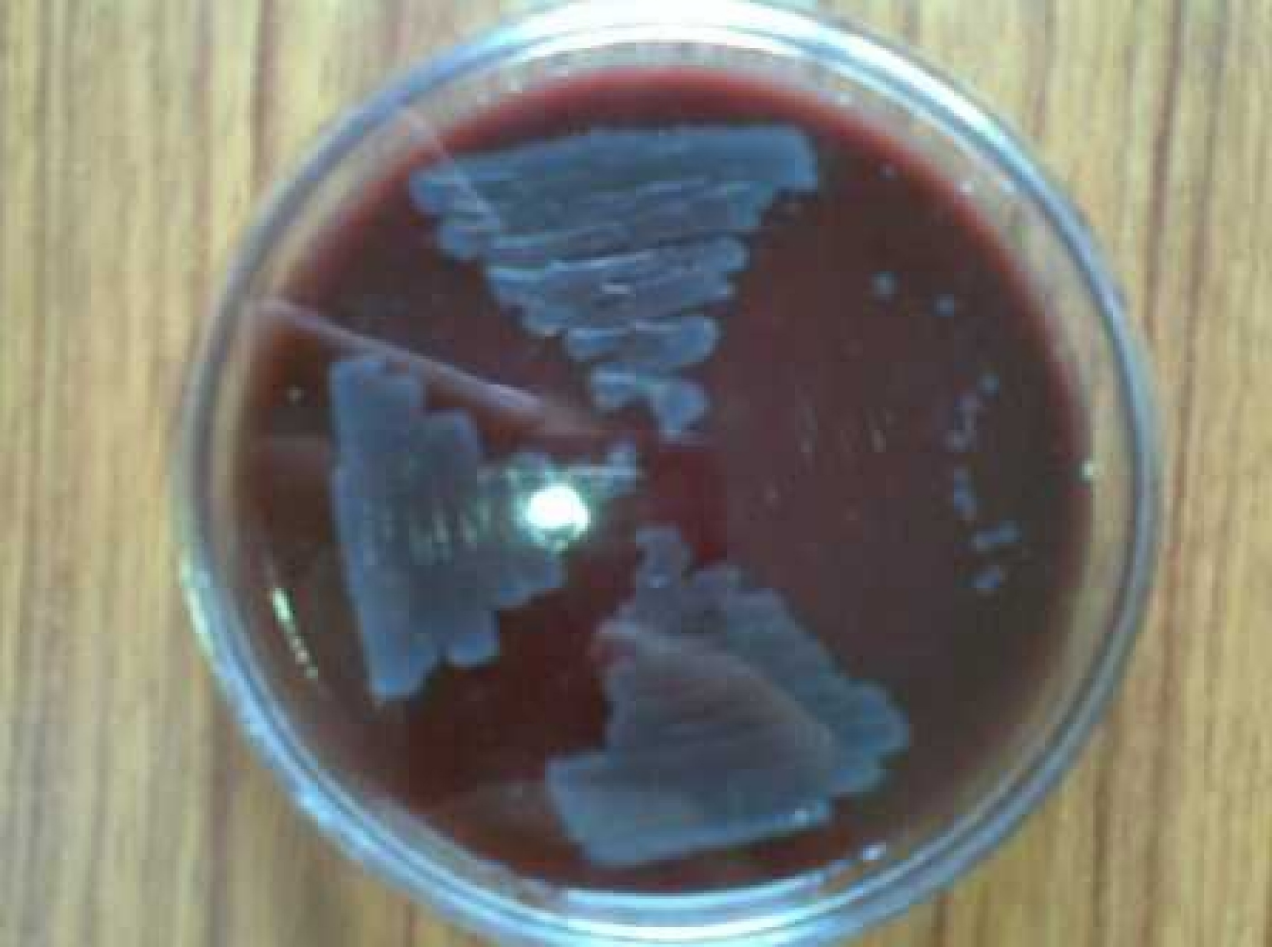
Serum Sensitivity and Resistant Pattern of E. coli

In the study group, cell surface hydrophobicity was observed in 9% of *E. coli *isolates, whereas in controls 10 (20%) strains exhibited cell surface hydrophobicity. In the study group, 93% of *E. coli *isolates were serum resistant and only two (4%) isolates showed serum resistance as shown in Figure 2.

**Figure 3:**
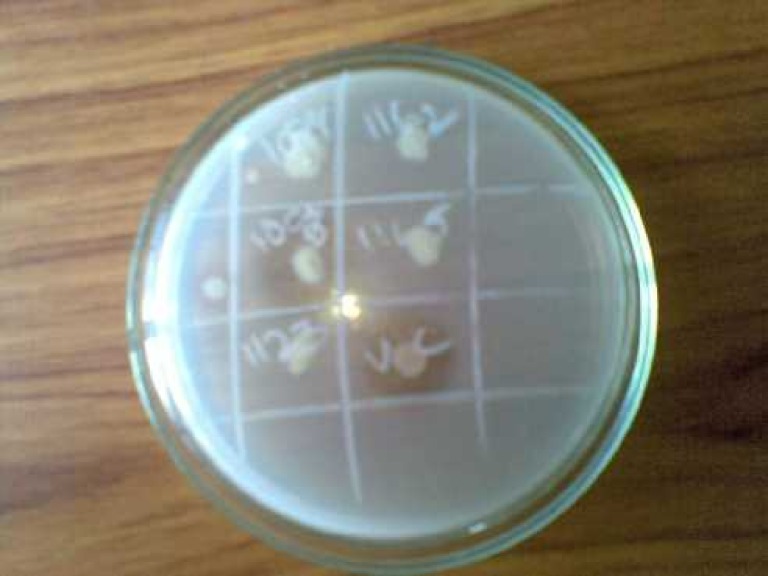
Gelatinase Production as Evidenced by Clearance Surrounding the Colonies

The study also showed that 2% of *E. coli *isolates were gelatinase producers and none among the control group demonstrated this virulence factor as shown in Table 3 and Figure 3.

**Table 3:** Comparison of Various Virulence Factors in Both Test Isolates and the Controls

Isolates	Hemolysin Production		Cell Surface Hydrophobicity		Serum Resistance		Gelatinase Production	
	Positive n (%)	Negative n (%)	Positive n (%)	Negative n (%)	Positive n (%)	Negative n (%)	Positive n (%)	Negative n (%)
Test strains	9 (9)	91 (91)	9 (9)	91(91)	93 (93)	7 (7)	2 (2)	98 (98)
Control isolates	0 (0)	50 (100)	10 (20)	40 (80)	2 (4)	48 (96)	0 (0)	50 (100)

**Figure 4:**
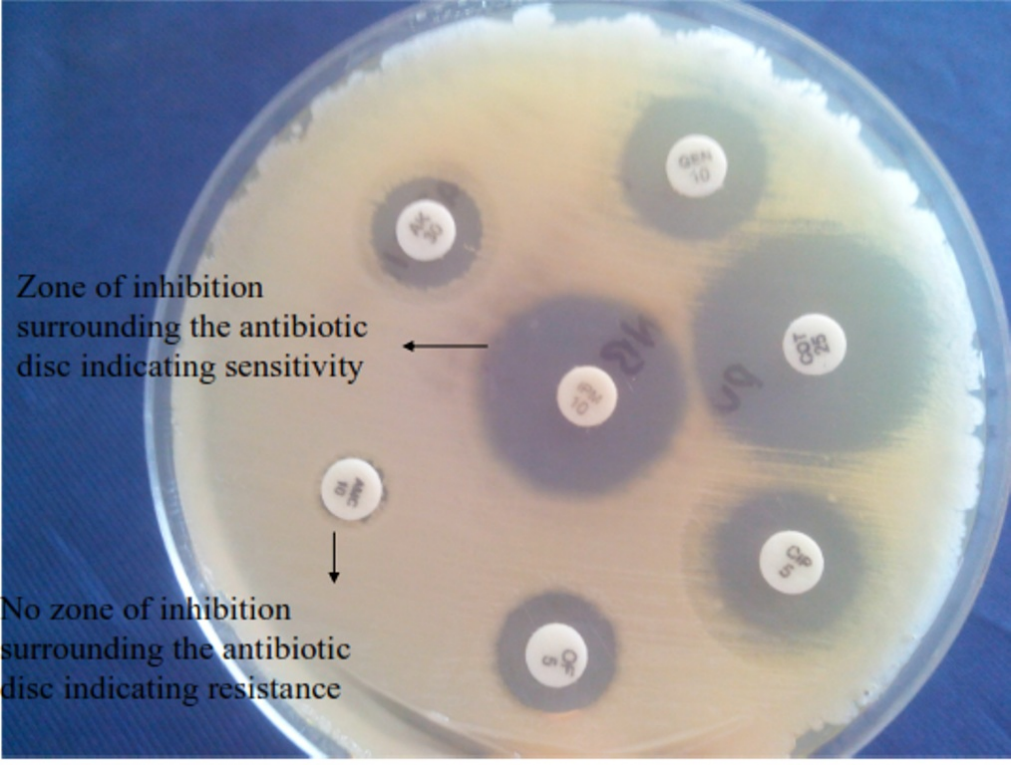
Antibiotic Susceptibility Plate Showing Zones of Clearance Surrounding Antibiotic Discs

Out of 100 *E. coli* included in the study only 65 strains were isolated from urine and among these isolates 62 isolates were found to be susceptible to nitrofurantoin and three showed resistance. Among test strains majority of *E. coli* isolates (80%) were sensitive to amikacin whereas only 47% of isolates were susceptible to co-trimoxazole. Susceptibility to ciprofloxacin/norfloxacin (22%) and ceftriaxone (28%) was noted to be low. A higher rate of resistance was observed against cephalothin (84%) and ampicillin (98%). None of the isolates from test strains showed resistance to imipenem. Among control strains included in the study, 46 (92%) isolates showed sensitivity to amikacin, 28 (56%) isolates revealed sensitivity to ciprofloxacin, and all isolates were sensitive to imipenem. The detailed antibiotic susceptibility profile of all the strains tested is shown in Table 4 and demonstrated in Figure 4.

**Table 4:** Antibiotic Susceptibility Patterns of Test and Control Strains of E. coli

Drugs	Sensitive		Intermediate		Resistant	
Type of strain	Test	Control	Test	Control	Test	Control
Ampicillin	1	-	1	-	98	50
Amikacin	80	46	4	2	16	2
Cotrimoxazole	47	-	-	-	53	-
Nitrofurantoin (only for urine isolate)	62	-	-	-	3	-
Cephalothin	9	10	7	-	84	40
Ceftriaxone	28	-	1	-	71	-
Ciprofloxacin/ Norfloxacin	21	28	-	-	79	22
Imipenem	100	100	-	-	-	-

## Discussion

*Escherichia coli* is a diverse group of bacteria, demonstrating variations both phenotypically and genotypically. Only 20% genetic similarity was observed between the many prevailing strains (a strain represents a subgroup within the species) of *E. coli*. *E. coli* strains are also subdivided based on the presence of surface antigens that include the somatic O antigen, the flagellar H antigen and the capsular K antigen. More than 150 serotypes of *E. coli* have been identified and related to different infectious conditions. The disease caused by a particular strain of *E. coli* depends on the type of virulence determinants it possesses as evidenced by the fact that some strains of *E. coli* cause mild diarrhea among adults and the same strains may produce a potentially life-threatening illness when they infect children/neonates. Virulence factors play an important role for *Escherichia coli *to colonize selectively the mucosal uro-epithelium, and starting an inflammatory reaction which helps it in proceeding from lower urinary tract to renal tissues. The capacity of *E. coli *to produce many virulence factors contributes to its pathogenicity and the ability to cause serious infections that include bacteremia and neonatal meningitis. Among controls none of the isolates exhibited all the four virulence factors and 38 (76%) *Escherichia* *coli* isolates have not revealed any of these virulence factors. All the isolates from the test group exhibited either one or more virulence factors suggesting the fact that the ability to cause infection improves with the presence of virulence factors.

Among the various determinants of virulence in invasive strains, α-hemolysin (HlyA), which hemolyzes red blood cells by forming pores in the erythrocyte membrane assumes significance. The frequency by which hemolytic *E. coli* strains can be isolated from patient samples increases with the severity of disease [13-14]. In the present study hemolytic activity was seen in nine percent of the cases and in none of the controls. Blanco et al. reported 32% hemolysin production among the cases which is a contrast to the results of the present study [15]. Kausar et al. in their study noted 21% hemolytic activity among test strains [16]. Raksha et al. have reported 41.36 % strains showing hemolytic activity among strains isolated from extraintestinal infections, which is way too high when compared to our study. The same study had reported six percent hemolytic activity among the control group i.e. fecal samples from apparently healthy people [8].

Cell surface hydrophobicity plays a key role in mediating bacterial adherence to mammalian cells. The crystalline surface layer ‘s’ present on both gram-negative and  gram-positive organisms plays a role in this hydrophobicity [9]. In the present study, nine percent of the strains were hydrophobic. Interestingly 10% of the control isolates too showed hydrophobicity. Blanco et al. in their research have reported 35% of isolates as demonstrating hydrophobic activity [15]. Bhat et al. reported 27.6% isolates to be hydrophobic in their study [2]. Raksha et al. in their study reported 26.4% strains demonstrating hydrophobic activity and found 10% of control strains to be hydrophobic, revealing a significant difference in statistics with regard to virulence detection [8]. 

Serum resistance is the property by which the bacteria resist killing by normal human serum due to the lytic action of the alternative pathway of complement system [17]. Bacterial resistance to killing by serum results from individual or combined effect of capsular polysaccharide, O polysaccharide, and surface proteins [18].

Isolates from patients with pyelonephritis, cystitis, and bacteremia were typically serum resistant whereas strains isolated from patients with asymptomatic bacteriuria were serum sensitive [18]. The serum-resistant gram-negative bacteria were found to possess a significant survival advantage in the blood during bacteremia [19]. In the present study, 93% of the isolates were resistant to serum bactericidal activity, whereas only four percent were serum resistant among control strains. The majority of the test strains which were positive for serum resistance were from urine (62 from a total of 65 urine samples) and pus (25 from a total of 29 pus isolates). Siegfried et al. have reported serum resistance in 68% of *E. coli* strains [10]. Raksha et al. in their research showed 32.7% of extraintestinal isolates and 24% of control strains to be resistant to serum bactericidal activity [8]. Our study results were comparable with studies done by Bhat et al. where they found that 86.8% of *E. coli *isolates showed serum resistance [2].

Gelatinase, an important virulence factor which is capable of hydrolyzing gelatin, collagen, and other bioactive peptides is associated with inflammation [20]. A study by Bhat et al. reported 6.9% gelatinase production whereas the present study detected gelatinase producing ability in only two percent of the isolates [2].

The present study revealed expression of multiple virulence factors by extraintestinal *E. coli *isolates. Most of the serum-resistant isolates were also hydrophobic, hemolytic, and gelatinase producers. In our study, none of the isolates showed the presence of all the four virulence factors. Presence of three virulence factors was noted in one percent isolates and the presence of two virulence factors was noted among 14% isolates. Studies have indicated that although virulence of an organism cannot be accurately predicted on the basis of its measurable virulence factor phenotype, the presence of multiple virulence factors does increase the virulence of organisms. 

The emergence of multidrug-resistant organisms restricts the choices for therapy for hospital-acquired infections [21]. Antibiotic susceptibility pattern was studied for all the isolates of* E. coli*. Majority of the isolates (80%) were sensitive to amikacin, 47% were sensitive to co-trimoxazole, and 93% of urine isolates were sensitive to nitrofurantoin (total of 65 urine isolates). Resistance was observed against various commonly used antibiotics such as ampicillin, nalidixic acid, cephalothin, ciprofloxacin/norfloxacin, and ceftriaxone. The presence of multi-drug resistance may be related to the dissemination of antibiotic resistance among hospital isolates of* E. coli*. Kausar et al. in their study have reported 92% strains as sensitive to amikacin, 85% sensitive to nitrofurantoin, 29%-45% sensitivity to co-trimoxazole, cephalothin, ciprofloxacin/norfloxacin, and ceftriaxone [11]. Another study done by Oteo et al. in their research reported resistance to ampicillin, cotrimoxazole, ciprofloxacin, gentamicin, and tobramycin at the rate of 59.9%, 32.6%, 19.3%, 6.8%, and 5.3% respectively [22]. These results support the hypothesis that various virulence factors and antibiotic resistance may confer increased fitness on the part of *E. coli* to cause extraintestinal infections in humans.

## Conclusions

The present study results re-establish the fact that *E. coli* strains are present in human, animal, and the environment and that most *E. coli* isolates are opportunistic pathogens. *E. coli* isolates from human extraintestinal infections have demonstrated the presence of various virulence factors which were absent in the *E. coli* strains present as commensals. *E. coli *has the ability to adapt and survive in humans by producing virulent factors and developing antimicrobial drug resistance. The virulence factor(s) could be selective and their expression may be related to the area of colonization.

Most virulence determinants are genetic and are phenotypically expressed only under certain conditions that include and are not limited to environmental factors and impaired host defence mechanisms. Identification of the ability of a bacterial strain to phenotypically express virulence factors and the related mechanisms could contribute to improved patient management. Since antimicrobial drug resistance is high among the *E. coli *strains isolated from human infections, a regular evaluation of antimicrobial susceptibility patterns of various clinical isolates and proper selection of antibiotics is warranted.
